# Comprehensive evaluation of the high‐resolution diode array for SRS dosimetry

**DOI:** 10.1002/acm2.12696

**Published:** 2019-09-03

**Authors:** Saeed Ahmed, Geoffrey Zhang, Eduardo G. Moros, Vladimir Feygelman

**Affiliations:** ^1^ Department of Radiation Oncology Moffitt Cancer Center Tampa FL USA; ^2^ Department of Physics University of South Florida Tampa FL USA

**Keywords:** diode arrays, high‐resolution array, multiple‐target SRS, SRS QA

## Abstract

A high‐resolution diode array has been comprehensively evaluated. It consists of 1013 point diode detectors arranged on the two 7.7 × 7.7 cm^2^ printed circuit boards (PCBs). The PCBs are aligned face to face in such a way that the active volumes of all diodes are in the same plane. All individual correction factors required for accurate dosimetry have been validated for conventional and flattening filter free (FFF) 6MV beams. That included diode response equalization, linearity, repetition rate dependence, field size dependence, angular dependence at the central axis and off‐axis in the transverse, sagittal, and multiple arbitrary planes. In the end‐to‐end tests the array and radiochromic film dose distributions for SRS‐type multiple‐target plans were compared. In the equalization test (180° rotation), the average percent dose error between the normal and rotated positions for all diodes was 0.01% ± 0.1% (range −0.3 to 0.4%) and −0.01% ± 0.2% (range −0.9 to 0.9%) for 6 MV and 6MV FFF beams, respectively. For the axial angular response, corrected dose stayed within 2% from the ion chamber for all gantry angles, until the beam direction approached the detector plane. In azimuthal direction, the device agreed with the scintillator within 1% for both energies. For multiple combinations of couch and gantry angles, the average percent errors were −0.00% ± 0.6% (range: −2.1% to 1.6%) and −0.1% ± 0.5% (range −1.6% to 2.1%) for the 6MV and 6MV FFF beams, respectively. The measured output factors were largely within 2% of the scintillator, except for the 5 mm 6MV beam showing a 3.2% deviation. The 2%/1 mm gamma analysis of composite SRS measurements produced the 97.2 ± 1.3% (range 95.8‐98.5%) average passing rate against film. Submillimeter (≤0.5 mm) dose profile alignment with film was demonstrated in all cases.

## 1 INTRODUCTION

Small malignant brain lesions are frequently treated with intracranial stereotactic radiosurgery (SRS) to obtain local control.^1^, ^2^ Intracranial radiosurgery is a complex, high‐precision procedure requiring submillimeter accuracy of the dose placement. That necessitates meticulous commissioning and ongoing quality assurance.^3^ Recently, an additional level of complexity was reached with the introduction of the single‐isocenter multitarget treatments.^4^, ^5^, ^6^, ^7^ Those plans often produce a large number of small multileaf collimator (MLC) apertures, and dosimetric commissioning and ongoing patient‐specific end‐to‐end tests^3^ require high‐resolution planar or volumetric detectors. Radiochromic film and gel/polymers have the required spatial resolution and have been employed for validation of the SRS techniques.^8^, ^9^, ^10^, ^11^ However 3D radiochromic dosimetry is too labor‐intensive and expensive for routine patient‐specific end‐to‐end tests.^12^ Even radiochromic film has its significant drawbacks, as the readout is delayed and quality dosimetry requires meticulous and time‐consuming calibration and readout protocols.^13^, ^14^, ^15^ Electronic detector arrays are in many aspects an attractive alternative but historically did not have sufficient spatial resolution for SRS measurements. Among the commercially available instruments, the first high‐resolution detector was an array of liquid‐filled ionization chambers detectors, with 2.5 mm detector size and pitch.^16^

In this work, we introduce a different planar array, designed primarily for SRS measurements in combination with a dedicated phantom. It consists of small diodes (essentially point detectors) with a 2.5 mm pitch and is a high‐resolution extension of the MapCHECK^17^, ^18^, ^19^ series of dosimeters (Sun Nuclear Corp., Melbourne, FL). Diode array readings generally exhibit dependence on the multitude of the radiation beam characteristics, and thus require an application of a sophisticated and rigorous calibration and correction formalism tailored to the individual design.^20^ We endeavored to validate the array’s individual basic calibration and correction parameters, as well as its performance in a series of end‐to‐end SRS‐type tests.

## 2 MATERIALS AND METHODS

### 2.1 Radiation sources

For logistical reasons, the measurements were performed on two TrueBeam linear accelerators producing conventional (6MV) and flattening filter free (6MVFFF) radiation beams. The beam energies from both machines were closely matched. Most basic array parameters were measured on the unit equipped with a standard 120‐leaf Millennium MLC with the leaves 5 mm wide as projected to isocenter. The SRS plans were delivered on the machine with the high‐definition (HD) MLC (2.5 mm wide leaves at isocenter).

### 2.2 Diode array and phantom

SRS MapCHECK (SMC, Sun Nuclear Corp, Melbourne, FL) consists of 1013 point (0.48 × 0.48 mm^2^ cross‐section, 0.007 mm^3^ active volume) diode detectors arranged on the two 7.7 × 7.7 cm^2^ printed circuit boards (PCBs). The PCBs are aligned face to face in such a way that the active volumes (p‐n junction) of all diodes are in the same plane. The detectors on the main board are facing up in the normal horizontal position. The spacing between the neighboring detectors on each board is 3.5 mm. However, the daughter board is shifted 1.75 mm relative the main board in both *X*‐ and *Y*‐axes, resulting in an overall inter‐detector spacing of 2.47 mm. The buildup and backscatter to the detectors are provided by 2.2 cm thick poly methyl methacrylate (PMMA) plates. According to the specifications, the device can handle the maximum repetition rate of 3400 MU/min, which exceeds typical values at isocenter for the FFF 6 or 10 MV radiosurgical beams.

**Figure 1:**
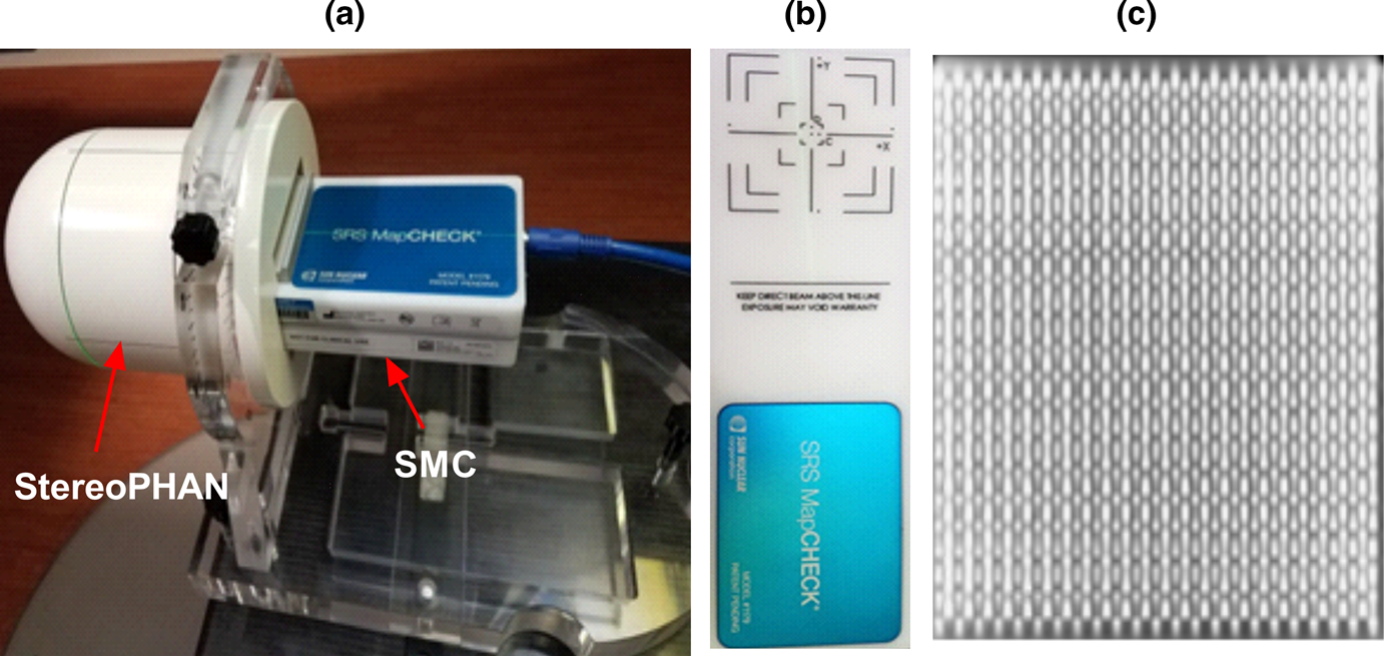
(a) SRS MapCHECK assembled with StereoPHAN, (b) SRS MapCHECK top view, (c) A CT‐based reconstruction of the 7.7 × 7.7 cm^2^ diode array.

StereoPHAN (Sun Nuclear) is specifically designed to accommodate the SMC for the end‐to‐end dosimetric testing of the SRS treatment plans. It is a cylindrical PMMA phantom with a hemisphere‐shaped rounded superior end, to mimic the head (Fig. 1). The diameter of both cylindrical and hemispherical parts is 15.24 cm and the total phantom length is 20.87 cm. The phantom has an inner 17.5 × 8.5 × 8.5 cm^3^ cavity. With appropriate spacers, this cavity accommodates the SMC as well as other imaging and dosimetric inserts, including those for ion chambers, radiochromic film, and special detectors (scintillator). The film insert houses a square 7.5 × 7.5 cm^2^ piece of radiochromic film. There are five embedded titanium fiducials to assist in aligning the phantom with onboard imaging and subsequent spatial registration of the film. The physical depth of the SMC detector active volumes, ion chamber center, and film plane inside the StereoPHAN is 7.62 cm. The distance from the superior spherical end of the phantom to the SMC central detector is the same. When aligned at the accelerator isocenter, the assembly (SMC inserted in the StereoPHAN) provides a means of true composite measurements with gantry and couch rotations. For the noncoplanar beams, the system supports the couch angles of up to ± 45°, to avoid direct irradiation of the array’s electronic by the primary beam.

### 2.3 Basic detector properties

#### 2.3.1 Calibration factors and corrections

To convert the raw diode readings to dose, a number of calibration factors and corrections have to be applied during the postprocessing stage. Those are derived or rectified during the calibration process. The user then has a choice in the control software (SNC Patient v. 8.0, Sun Nuclear) to process the measurement with specific corrections turned on or off.

The phantom assembly should be represented in the treatment planning system (TPS) as a homogeneous body, as the calibration process removes the response dependence on the internal structure of the device, much like an ion chamber calibration removes the influence of the chamber design. All basic tests were performed for both 6MV and 6MVFFF beams.

*The absolute calibration* is applied to the master (central) diode. It was derived from the known calibration dose value from the TPS calculation at the diode location in an open field.

*The relative array calibration* (response equalization) accounts for the inherent differences in the diodes’ sensitivity. It generally follows the standard wide field procedure of sequential irradiations in a conventional (flattened) beam with array shifts and rotations.^21^, ^22^ However the procedure is somewhat different from the previously described original MapCHECK calibration.^17^, ^18^ Because of the two PCB with detectors facing in opposite directions, the sequence is repeated with the array facing towards and away from the beam (“AP and PA” calibrations). This calibration is performed in the manufacturer‐supplied PMMA slab phantom. Also, for the optimal measurement accuracy with FFF beams, two additional measurements in the StereoPHAN are required: “AP and PA” 5 × 5 cm^2^ fields. In addition to response equalization, the measurements in the parallel‐opposed fields help with scaling the angular response function described later.

The effectiveness of the relative calibration is typically verified by a 180° array rotation test. To that end, two identical exposures in a vertical wide field (10 × 10 cm^2^) were delivered with the SMC electronics facing either the foot or the head of the couch. Percent dose differences for each detector between the standard and rotation orientations were recorded.

*Angular dependence* is a well‐known phenomenon for the diode arrays of this type and must be corrected for in composite‐type measurements.^12^ The difference in response of nearby diodes on the two PCBs facing towards or away from the beam is appreciable and changes as a function of the beam incidence angle. This change serves as the basis for the incidence angle approximation. The previously measured angular response function, which should be for the most part relatively smooth, is scaled based on the “AP and PA” wide field calibration measurements.

The angular correction efficacy with gantry rotation was verified in the transverse (couch at 0°) and sagittal (couch at 90°) planes, as well as for the various combinations of couch and gantry angles, using standard methodology of measurements against detectors with nearly isotropic response.^12^ The phantom with the SMC was placed at the accelerator isocenter. The response was compared at the central axis (CAX) and at the off‐axis diodes against the 0.125 cc Semiflex ion chamber (PTW, Freiburg, Germany), and/or a 1 mm diameter, 3 mm long, water‐equivalent^23^, ^24^ scintillator detector (W1‐PSD, Standard Imaging Inc., Middleton, WI). The scintillator was calibrated for Čerenkov radiation discrimination with a minimum/maximum fiber length exposure in a solid water‐equivalent phantom, with the beam perpendicular to the long axis.^25^, ^26^ The SMC data were processed with and without applying the angular correction factors to distill the effect of the corrections.

For the *transverse plane angular dependence at CAX*, the field size was set to 5 × 5 cm^2^ and the gantry was rotated in 10° increments, which were progressively reduced to 2° and 1° as the beam direction came closer to the array plane. The SMC response was compared to the ion chamber for both energies. The same SMC data were also verified against the W1‐PSD for the 6MVFFF beam.

For *sagittal plane angular dependence at CAX* measurements, the couch was placed at 90° and the gantry rotated in 30° increments. The incidence angles where the beam could directly irradiate the electronics were avoided. The SMC results were evaluated against the W1‐PSD.

For the *combined couch and gantry rotations*, the couch angular positions were 0, ±10, ±30, ±50, ±70, and ±90°. At each couch position the gantry was rotated in 30° intervals. The data were again compared to the W1‐PSD.

In addition to the diode at the CAX, four *off‐axis points* located at the different locations in the SMC array were selected (Table 1). The measurements were performed with the combinations of nonzero gantry and couch angles for each point. The field size was 8 × 8 cm^2^. The SMC response for both energies was compared to the W1‐PSD. Point P4(−21,31.5) was used for couch rotations of 0 and 10°. For other couch angles, P4a(−21,21) was used instead to avoid positioning the detector in the penumbra.

**Table 1:** Measurement point coordinates (IEC *X* and *Y*).

Point	P0	P1	P2	P3	P4	P4a
*X* (mm)	0	31.5	10.5	−10.5	−21	−21
*Y* (mm)	0	21	31.5	10.5	31.5	21

The accelerator *repetition rate* (MU/min) dependence was previously reported for various Sun Nuclear devices^18^, ^20^, ^27^ and is corrected for in the SMC software. The correction is applied based on the measured pulse rate during the collection cycle (50 ms). Its efficiency was investigated for repetition rates ranging from 10 to 600 MU/min for conventional and 400 to 1400 MU/min for FFF 6MV beams. Field size was 5 × 5 cm^2^ and 100 MU were delivered at each repetition rate. The data were normalized to the ion chamber readings, which in turn showed negligible collection efficiency difference by the two‐voltage technique across the range of repetition rates. The SMC readings were processed with and without the repetition rate correction applied.

*Diode sensitivity dependence on field size* is also a well‐known phenomenon that needs to be accounted for to obtain accurate dosimetric results. The SMC software estimates the equivalent field size based on the number of diodes simultaneously registering above‐the‐background signal and applies an energy‐specific correction factor measured with radiochromic film for the field sizes down to 5 × 5 mm^2^. This correction formalism was verified against the W1‐PSD detector for square MLC‐defined fields ranging from 5 to 40 mm on a side. The scintillator was inserted in a specially designed rectangular solid water phantom and centered in the radiation field such that its long axis was parallel to the beam central axis (e.g., 1 mm detector cross‐section). The buildup thickness was chosen so that the effective point of measurement (taken to be the middle of the 3 mm active length) was at the same water‐equivalent depth as the central SMC diode in StereoPHAN. The SMC was also used outside of the StereoPHAN, while maintaining the same water‐equivalent buildup and backscatter. The angular corrections were disabled in the control software, since for the very small fields the system cannot determine the beam incidence angle and reverts to the average correction, which would have distorted these measurements. The SMC field size dependence was studied with both “AP and PA” beams.

#### 2.3.2 Response linearity with monitor units

For completeness, dose response linearity with monitor units was investigated. The monitor units for a 6MV beam varied from 1 to 100, and the SMC response was compared to the ion chamber. The diode readings were averaged between five centrally located detectors (e.g., within a 3.5 × 3.5 mm^2^ square).

### 2.4 End‐to‐end tests

#### 2.4.1 Treatment planning

The device is intended to be used for the true composite (e.g., with planned gantry and table angles)^28^ SRS measurements. The tests involved the dosimetric comparison of the SMC measurements against the radiochromic film for three SRS treatment plans for each energy. The StereoPHAN was scanned on a 16‐slice CT scanner (Philips Medical, Cleveland, OH) according to our standard SRS protocol (sequential scans with 1.25 mm slice thickness). Four CT datasets were acquired with different inserts to facilitate accurate placement with on‐board kilovoltage imaging.

**Figure 2:**
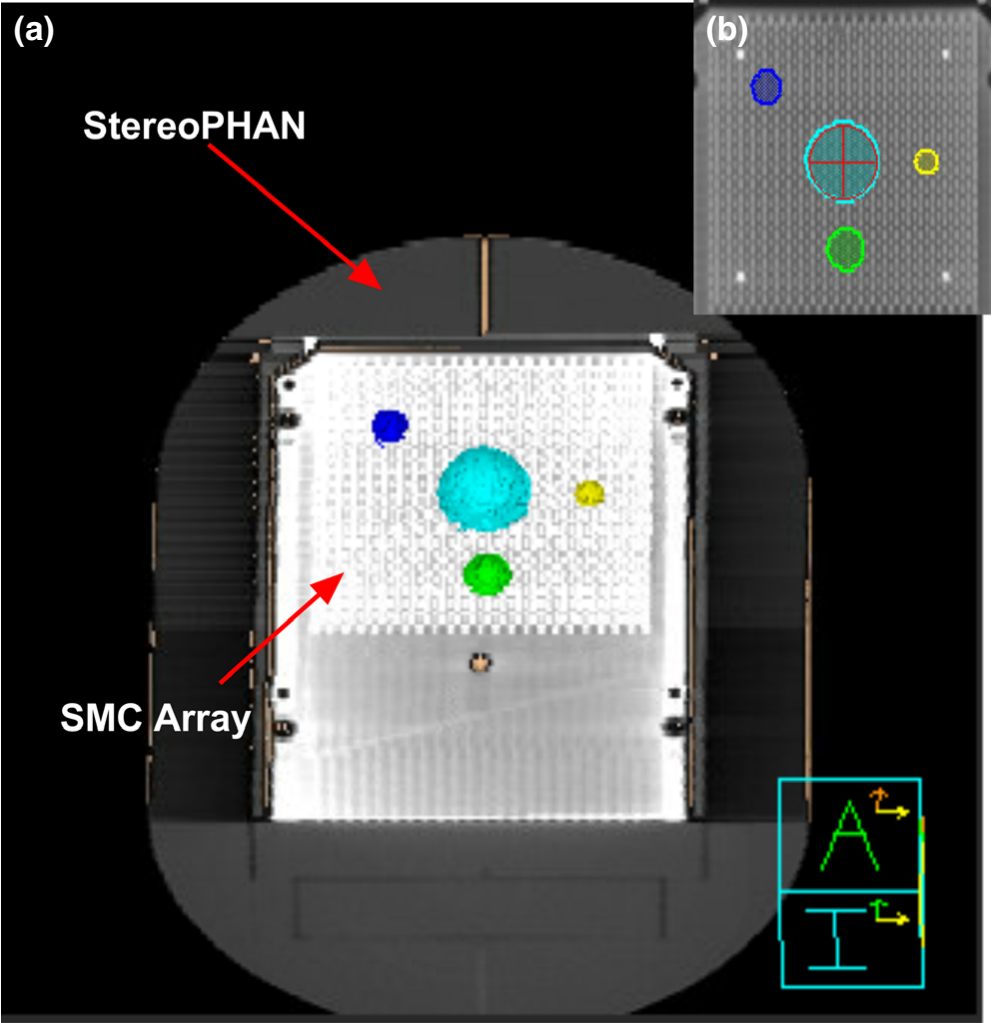
The arrangement of spherical targets superimposed (a) on the SMC array, and (b) on the film fixture. The film fixture image also includes the diode array reconstruction fused to it.

Treatment plans were developed using Pinnacle v. 16.0 (Philips Radiation Oncology Systems, Fitchburg, WI). The isocenter was placed based on the phantom marks and film fiducials visible on the CT scans. Three multiple‐target plans were created, each with three spherical targets bisected by the coronal plane and situated within the active area of the array. Each plan had targets of different sizes randomly placed at the different locations. The target diameters ranged from 0.5 to 1.3 cm. Fig. 2 provides an example of the target arrangement around the isocenter. The plans were optimized to deliver conformal high dose to each target while minimizing dose to the remainder of the volume, using a single‐isocenter VMAT technique. The details of the plans are provided in Table 2. The VMAT optimization employed two full coplanar and two partial (130° or 90° span) noncoplanar arcs. The last two columns in Table 2 provide the details of the couch and gantry angles for each plan. Each target was planned to receive 24 Gy.

**Table 2:** Treatment plan details.

Plan	No. of targets	Target max Dimensions (cm)	Target Center distance from isocenter (cm)	Max field size, *X* × *Y* (cm^2^)	Couch angles	Arcs full/partial
1	03	1.0, 1.3, 0.5	2.7, 3.0, 3.3	7.5 × 8.0	±30°	2(356°)/2(130°)
2	03	0.7, 1.0, 1.2	3.2, 2.8, 3.1	8.2 × 7.8	±20°	2(356°)/2(130°)
3	03	0.6, 1.0, 0.8	2.5, 2.4, 3.0	7.1 × 7.3	±40°	2(356°)/2(90°)

In addition to the three targets, a 2 cm diameter spherical structure was drawn at the isocenter and was included in the optimization to achieve a low‐gradient 18 Gy dose region across the 0.125 cc chamber. This allowed us to more accurately convert film density to dose by applying an ion chamber‐derived scaling factor in addition to the calibration curve.

#### 2.4.2 Comparison measurements

All plans were delivered on a TrueBeam accelerator with a HD Millennium MLC and a six‐degrees‐of‐freedom (6DOF) couch. Each plan was delivered to the StereoPHAN three times with different detectors in place: the Semiflex ion chamber at the center, the SMC, and the film, with the latter two oriented in the coronal plane. Before each measurement, the phantom/detector were first leveled with a digital level for pitch and roll and then aligned for 3D shifts and yaw by cone‐beam CT with the help of the 6DOF couch. The ion chamber in the StereoPHAN was cross‐calibrated against the TPS calculated dose in a parallel‐opposed pair of 10 × 10 cm^2^ fields.

Extended range Gafchromic film (EBT‐XD, Ashland Inc., Bridgewaer, NJ) was used for the dosimetry of SRS plans. The EBT‐XD film has an optimum dose range of 0.4–40 Gy and has no significant dependence on energy, dose rate,^29^ and scanning orientation.^30^ The film calibration curve was obtained in the usual fashion in the range of 2–40 Gy.

Film processing followed a meticulous protocol paramount for accurate dosimetry.^10^, ^29^, ^30^ The film sheets were cut into 7.5 × 7.5 cm^2^ squares, marked to preserve the original orientation, and each piece was scanned individually to establish the background density map. An opaque cutout template was used to reproducibly position the film for scanning. All films were scanned using 48 bit color flatbed document scanner (Expression 11000 XL, Epson Seiko Corporation, Nagano, Japan) 24 h after the exposure, in transmission mode and without applying any software corrections. The pixel resolution was 72 dots per inch (0.35 mm/pixel).

The red channel^29^ images were processed in RIT software v. 6.6 (Radiological Imaging Technologies, Cololrado Springs, CO). The individual background correction maps, followed by the calibration curve were applied to each film. The absolute dose was further scaled to match the ion chamber dose at the isocenter.^28^ For scaling purposes, the film dose was averaged over 18 central pixels (~6.5 mm) in the craniocaudal direction, approximating the length of the Semiflex chamber active volume. The corresponding planar dose distributions from SMC and film were imported into RIT as a reference and target dose images, respectively. A template based registration was performed based on the phantom fiducials’ imprints on the film. The SMC and film measured doses were compared using three techniques. First, the gamma analysis^31^ was performed with two criteria combinations (3%/1 mm and 2%/1 mm) with global dose‐error normalization and low‐dose cutoff set at 10% of maximum dose. The RIT digital gamma analysis routine based on Depuydt et al^32^ was used. Second, the dose difference between the film and SMC measurements was evaluated in the peak‐dose/low‐gradient region near the center of each target. The dose was averaged over 5 pixels (1.75 mm). Finally, the spatial alignment of the SMC and film measured dose profiles was studied for every target. The in‐plane and cross‐plane (IEC *Y* and *X*) profiles were sampled through the center of each target on both the SMC and film dose maps. The profile center was defined as the middle of the full width at half‐maximum. For a few cases, the targets were too close to each other precluding the centering at the 50% dose levels, and they were aligned based on the 60% level. In total, 14 cross‐plane and 18 in‐plane profiles were examined.

## 3 RESULTS

### 3.1 180° Rotation test

For the wide field irradiation, the average percent dose‐error between the normal and rotated SMC response for all diodes was 0.01% ± 0.1% (range −0.31% to 0.4%) and −0.01% ± 0.17% (range −0.93% to 0.91%) for 6 MV and 6FFF MV beams, respectively.

### 3.2 Angular dependence in the transverse plane

**Figure 3:**
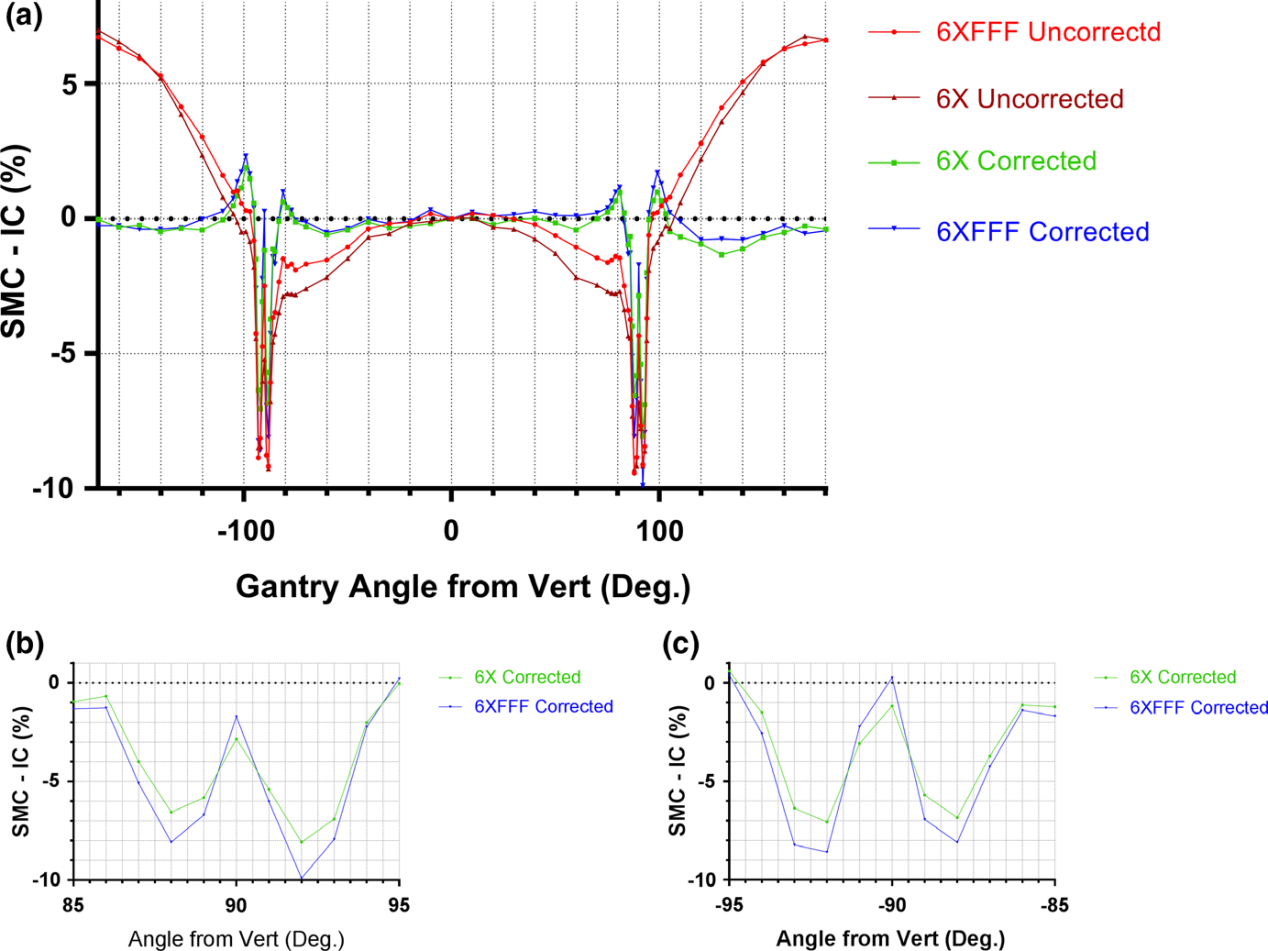
Angular dependence of the SMC central diode with couch at 0°; (a): Percent difference between SMC and ion chamber readings for the full 360° range; (b) and (c): A blow up of angular intervals close to ± 90°.

Fig. 3 shows the central diode response dependence on the gantry angle with the couch at 0°, compared to the ion chamber. The corrected response stays within 2% from the ion chamber for all gantry angles, until the beam direction approaches the detector plane (within ±5° of ±90° gantry angles) where deviations as large as −9% could be observed for both energies. The uncorrected response naturally shows larger deviations for a range of gantry angles. The 6MVFFF scintillator detector data were very close to the ion chamber results.

### 3.3 Angular dependence in the sagittal plane

**Figure 4:**
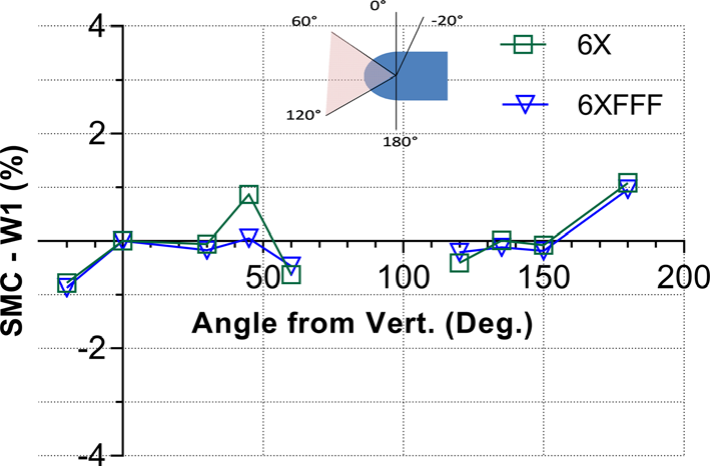
Percent difference between the SMC and scintillator detector response in the sagittal plane (couch at 90°) for both energies. The insert illustrates the solid angle interval from 60 to 120° for which the measurements could not be performed due to the direct beam exposure of the device electronics.

The response characteristics with the couch at 90° are presented in Fig. 4. The device agrees with the scintillator to within 1% for both energies.

### 3.4 Angular response with gantry and couch angle combinations for diodes at central and off‐axis locations

**Figure 5:**
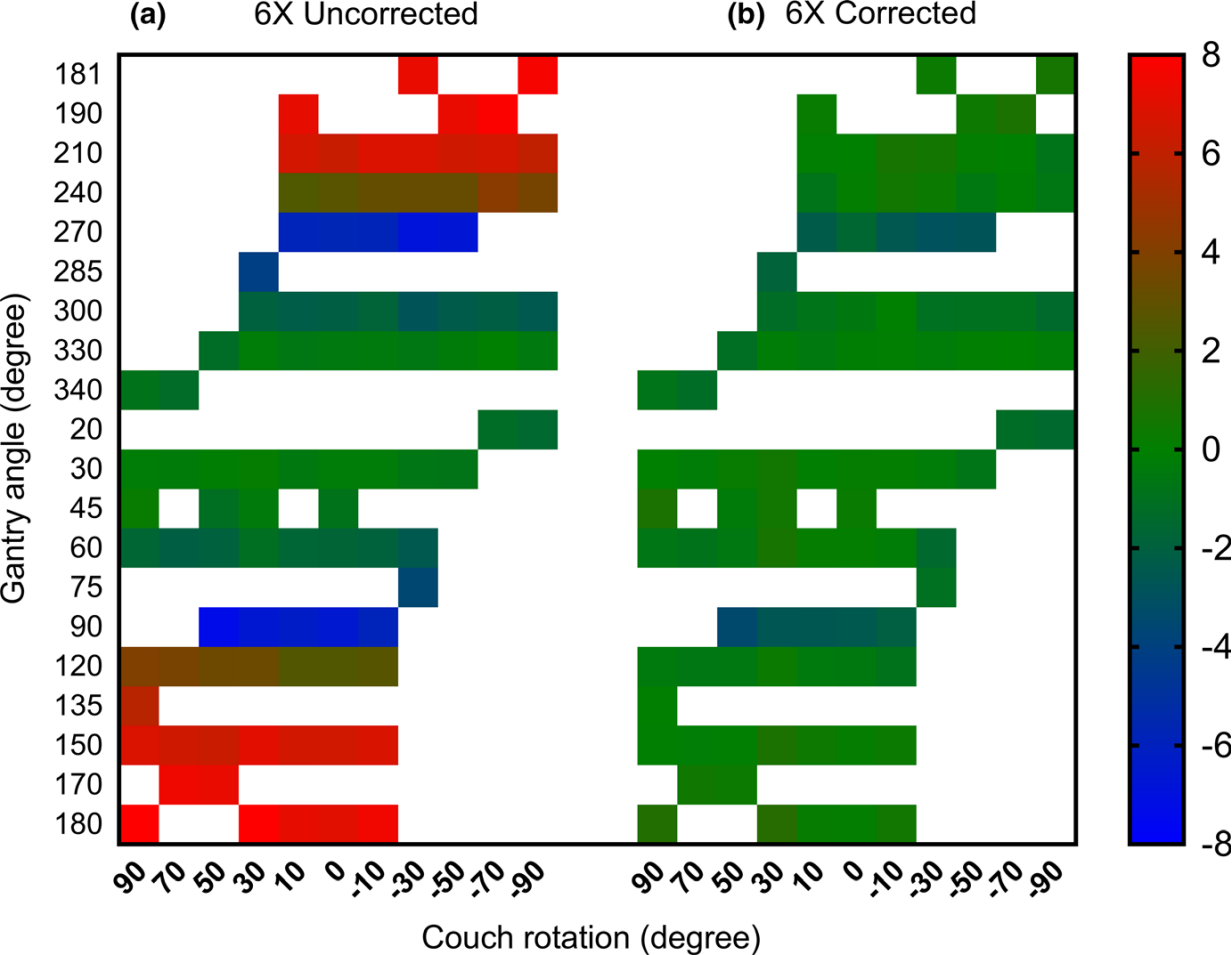
A heat map of SMC readings percent errors compared to the scintillator in a 6MV beam, for different combinations of gantry and couch angles.

The angular dependence of SMC response was further sampled for a range of combinations of gantry and couch angles. Fig. 5 shows the heat map of percent errors for uncorrected and corrected central diode response against the W1‐PSD for the 6MV beam. The uncorrected measurement data show deviations as large as 8% at the large gantry and/or couch angles. The corrected response, however, agrees with the W1 scintillator detector within 1% for all gantry and couch angles studied in this section, except for gantry angles at ±90° where the device was found under‐responding by ~3%. A similar behavior was observed for the 6MVFFF beam. (Table 3).

**Table 3:** Descriptive statistics of percent dose errors of diode located at the central axis and off‐axis locations vs W1 scintillator for all combinations of gantry and couch rotations in this section.

	Central diode		Off‐Axis diodes	
	6X	6FFF	6X	6FFF
Minimum	−3.5%	−1.8%	−2.1%	−1.6%
Maximum	1.2%	1.0%	1.6%	2.1%
Mean	−0.4%	−0.3%	−0.00%	−0.1%
SD	1.0%	0.6%	0.6%	0.5%
95%CI	−0.6% to −0.2%	−0.4% to −0.2%	−0.1% to 0.1%	−0.2% to −0.03%

**Figure 6:**
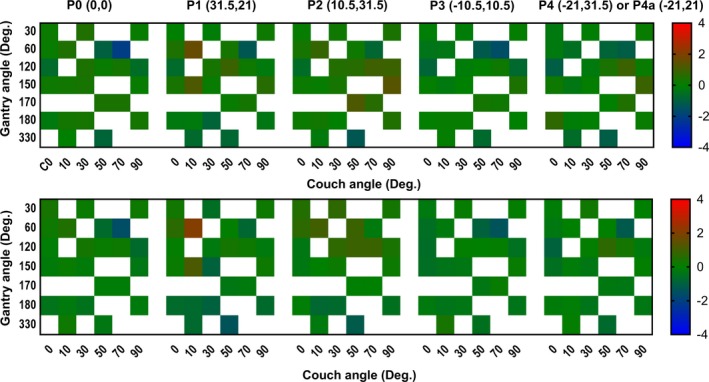
Percent errors for SMC off‐axis diodes r response (at points P0–P4 in Table 1) against the W1‐PSD. The upper graph shows the data for the 6MV and the lower one for the 6MVFF beam.

Fig. 6 shows the corrected response of the SMC off‐axis diodes compared to the W1‐PSD. The upper row shows the percent error heat map for the 6MV and lower for the 6MVFFF energies, respectively. The average percent errors were −0.00% ± 0.6% (range: −2.1% to 1.6%) and −0.1% ± 0.5% (range −1.6% to 2.1%) for the 6MV and 6MVFFF beams, respectively.

Table 3 lists the descriptive statistics for the percent errors for the diodes both at the CAX and off‐axis locations for both energies.

### 3.5 Repetition rate dependence

**Figure 7:**
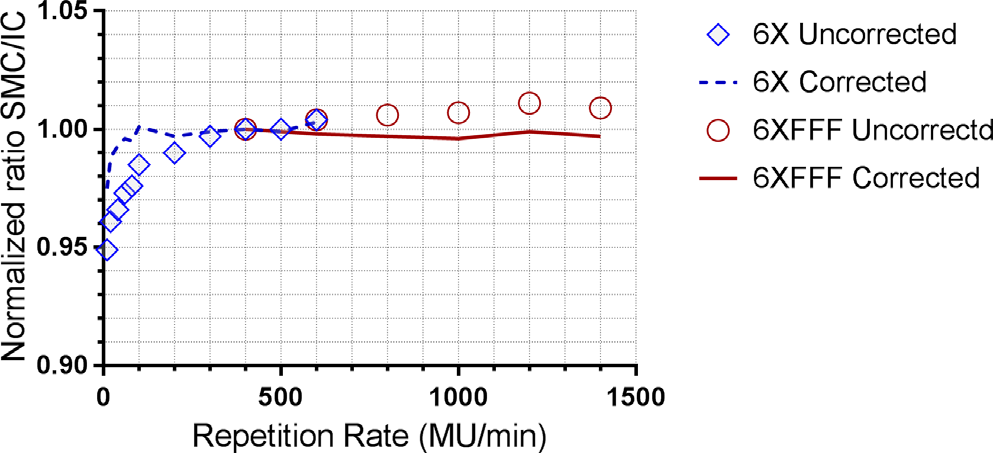
Repetition rate dependence of corrected and uncorrected SMC response compared to the ion chamber. For both energies the readings are normalized at a common value of 400 MU/min.

The repetition rate dependence is presented in Fig. 7. The SMC response with repetition correction applied agrees with the ion chamber to within 1% for 6MV and 0.5% for 6MVFFF for the repetition rate range studied. The maximum deviation of 2.5% was observed for 6MV beam at 10 MU/min.

### 3.6 Diode sensitivity dependence on field size

**Figure 8:**
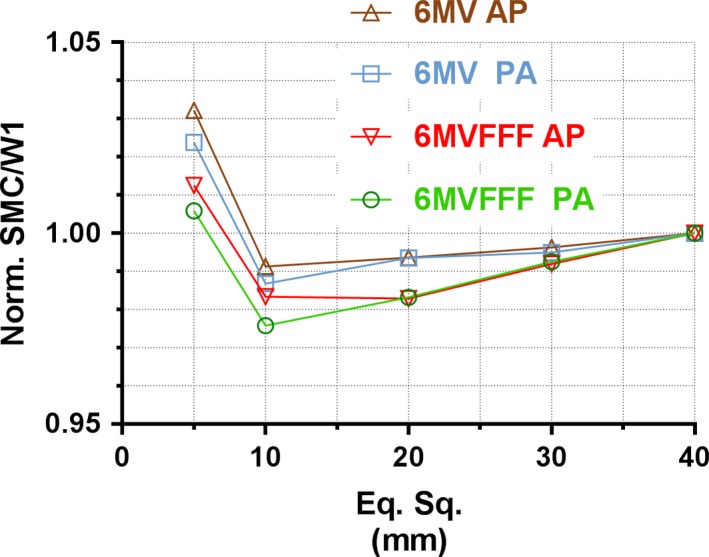
Ratio of relative output factor, SMC vs the W1‐PSD.

Fig. 8 presents the ratio of output factors measured with SMC and the W1‐PSD for both energies, normalized to a 40 × 40 mm^2^ field. The largest disagreement (3.2%) was seen for the 6MV 5 × 5 mm^2^ field. Otherwise the errors are largely within 2%. Both “AP and PA” beam orientations are included in the figure, and the corresponding readings differ by no more than 0.8 percentage points.

### 3.7 Response linearity with monitor units

**Figure 9:**
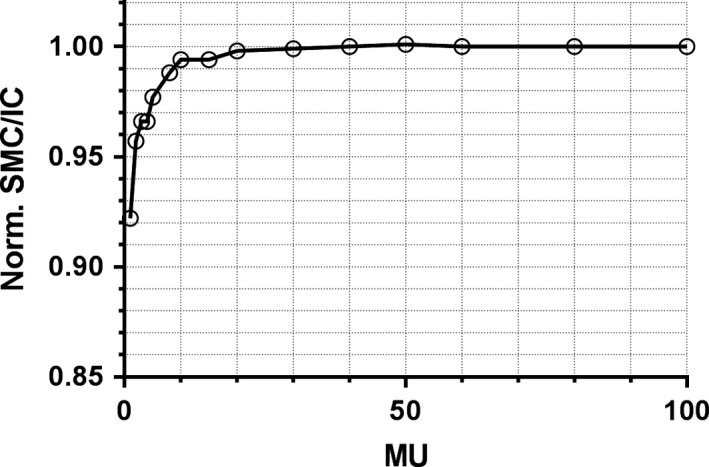
The ratio of SMC to ion chamber readings as a function of monitor units, normalized to 100 MU.

The SMC response relative to the ion chamber is plotted in Fig. 9. The difference is less than 2% upwards of 6 MU. After the sensitivity curve reaches a plateau, the higher monitor units are not a concern since the readout electrometers are reset after each 50 ms collection cycle.

### 3.8 End‐to‐end tests

#### 3.8.1 SMC vs Film gamma analysis

Table 4 shows the gamma analysis passing rates for SMC measurements against film.

**Table 4:** Gamma analysis passing rates: SMC vs film.

	γ‐Analysis pass rate (%)	
Plan	3%G/1mm	2%G/1mm
6MV_1	97.6	95.9
6MV_2	98.0	95.5
6MV_3	99.8	98.6
6MVFFF_1	99.5	97.5
6MVFFF_2	99.1	97.1
6MVFFF_3	99.6	98.5
Average	98.9	97.2
SD	0.9	1.3
95%CI	98.0‐99.9	95.8‐98.5

**Figure 10:**
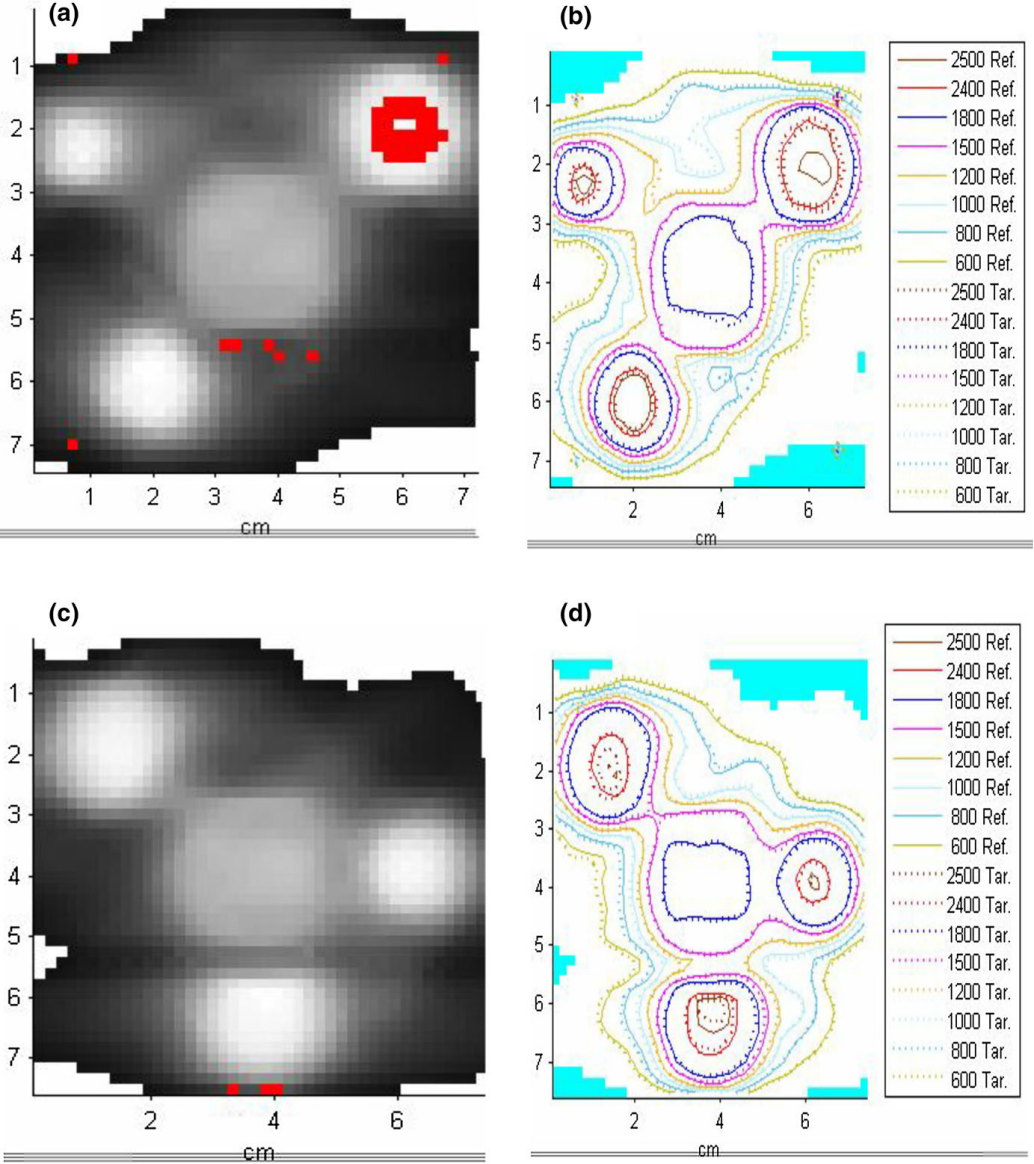
An example of gamma pass/failure maps (3%/G1mm) and isodose overlays (SMC vs film) for two plans: (a and b): 6MV_1 and (c and d): 6MV_3.

Fig. 10 illustrates the gamma pass/fail maps (3%G/1 mm) and the isodose overlays for two different plans. It provides a visual example of the worst and best agreement: plan 6MV_1 with the passing rate of 97.6% and 6MV_P3 plan with the passing rate of 99.8%. The pixels failing the gamma analysis are shown in red. The disagreement in the majority of plans is mostly confined to the lower dose regions (<10 Gy) although discrepancies inside a target were observed, which prompted a separate analysis of the peak dose agreement described in the next section.

#### 3.8.2 Profiles peak dose values and spatial alignment

Most of the SMC peak target doses were within 2% of the film measurements. The overall average percent dose error was −1.06% ± 1.82%, with the rest of the descriptive statistics summarized in Table 5. The peak dose‐error distribution passed the D'Agostino & Pearson normality test, that is, the null hypothesis that all values have been sampled from the normal distribution could not be rejected (*P* = 0.829). The differences in the center coordinates of the SMC and film profiles were all below 0.5 mm (Table 5).

**Table 5:** Descriptive statistics of the percent peak dose differences (ΔD) and the profile center misalignment for all plans and targets.

	ΔD (%)	Profile center (SMC‐Film) (mm)	
		X	Y
Minimum	−4.2	−0.5	−0.2
Mean	−1.06	0.1	0.01
S.D	1.8	0.3	0.1
Maximum	2.7	0.4	0.2
95%CI	−2.0% to −0.2%	−0.1 to 0.2	−0.04 to 0.1

For peak dose difference 18 targets were analyzed. For the profile centroid investigation in the cross‐ (*X*) and in‐plane (*Y*) orientations, 14 and 18 targets were sampled, respectively.

**Figure 11:**
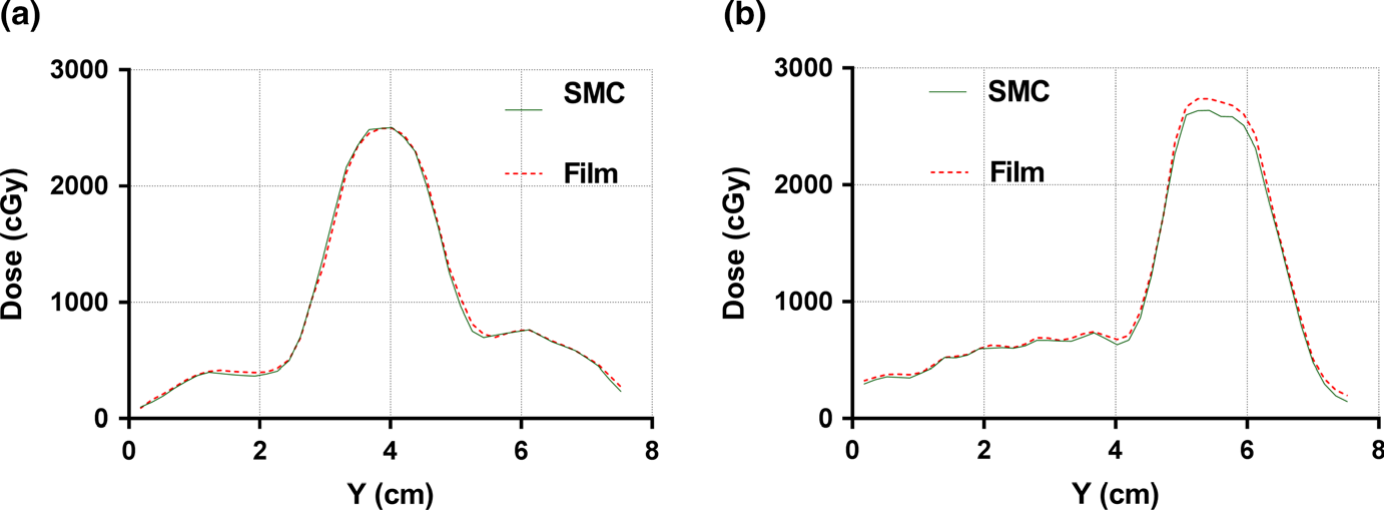
In‐plane profile comparison through the centers of the targets between SMC and film. (a) plan 6MV_3 (0.6 cm target T1) and (b) plan 6MV_2 (1.2 cm target T3).

Fig. 11 presents a visual example of in‐plane profile overlays for two targets, again corresponding to the best‐ and worst‐case scenarios.

## 4 DISCUSSION

Overall, the basic characterization tests indicated that the device performed within specifications and with sufficient accuracy. The 180° array rotation test resulted in an average error close to 0%, demonstrating successful diode response equalization. The repetition rate correction brings the response variation with MU/min to less than 1% at worst, in the range primarily relevant to radiosurgery. In the study of the field size dependence, the SMC readings tend to overrespond, compared to the scintillator, for the smallest measured field (5mm) and under‐respond between 10 and 40 mm. Overall, the agreement is reasonable. With the exception of the 5 mm field at 6MV (3.2% error) the difference from the scintillator is within 2.4% or better. Results with opposite beam orientations with respect to diodes are within 0.8 percentage points from each other. The corrected axial angular response did not deviate from the two nearly isotropic dosimeters by more than 2% for the wide range of gantry angles, except for the narrow angular intervals when the beam direction is (nearly) parallel to the array plane. This is typical for all arrays and has relatively small effect on the composite measurements.^12^, ^33^ The purely azimuthal dependence did not exceed 1%. To conclude the open filed measurements, for a large number of combinations of gantry and couch rotations the mean deviation from a small water‐equivalent detector did not exceed 0.4%, with the 95% confidence interval within 0.6% of zero at worst.

The basic dosimetric properties of the device were also verified for the 10 MV FFF beam, although the detailed results are not presented for brevity. Briefly, since the same correction methodology was used for the higher energy, the results are very similar to the 6 MV beam. Notably, the response did not deviate from the ion chamber by more than 0.5% with the repetition rate up to the maximum 2400 MU/min. The field size dependence was corrected to within 1% compared to the scintillator for all field sizes. The angular response was studied for the central diode against the IC and followed the same pattern as for the 6 MV beam, with the deviation not exceeding 2% except when the beam was nearly parallel to the detector plane.

There are two other possible factors that can influence the dose reported by a diode. One is temperature,^34^ which is accounted for in the device with the help of multiple sensors on the PCBs. This correction’s most important function is to compensate for the temperature gradient across the array caused by the normal heating of the electronics module. It was not explicitly evaluated, although it is implicit in the response equalization results. Another one is dose‐per‐pulse dependence,^17^ which was ignored, since the detector is designed to be placed at the isocenter with the minimal variation in dose‐per‐pulse between calibration and measurements.

During the end‐to‐end testing with multiple‐target single‐isocenter noncoplanar VMAT plans, fairly high gamma analysis passing rates between the SMC and radiochromic film dose distributions were observed with both 3%/1mm and 2%/1mm criteria combinations, particularly taking into account the gafchromic film accuracy estimated at 2–3%. For the intended applications of the array it was important to keep the distance to agreement criteria low, at 1 mm. The peak (target) dose‐error distribution cannot be statistically distinguished from normal. However, there appears to be a small bias of the mean, with SMC under responding by 1.1 ± 1.8% (1SD). The precise cause of this bias was not ascertained, but we noticed that both the field size dependence and angular dependence at near‐parallel incidence errors in most cases have the same sign consistent with the observed bias direction. Finally, of paramount importance in SRS, the film and SMC peak dose profiles coincide and show submillimeter distance to agreement for every target in every plan. This is consistent with the notion that a grid of point detectors with 2.5 mm spacing is sufficient, in terms of Nyquist sampling, to faithfully represent dose gradients encountered in megavoltage radiotherapy.^35^, ^36^ That was experimentally confirmed once again with the 2D liquid‐filled ionization chamber array having the detector size and pitch of 2.5 mm.^16^

In terms of the array’s utility for clinical SRS dosimetry, it can be characterized as a sufficiently accurate and convenient tool for commissioning an SRS beam, including simultaneous multitarget treatments. During the commissioning, the artificial targets can be fairly easily moved around to coincide with the active area. The device is well suited for single‐target patient‐specific end‐to‐end testing recommended for SRS treatments.^3^ Its application can be logistically more challenging for single‐isocenter multiple‐target plans. To sample all the targets, multiple measurements at different array angular orientations might be necessary. In addition, some targets could be farther away from the isocenter than the extent of the active area. As an alternative, supplementing/replacing direct measurements with semiempirical 3D dose reconstruction could be considered for such cases.^10^

## 5 CONCLUSIONS

The SRS MapCHECK diode array in the StereoPHAN phantom has sufficient dosimetric accuracy and spatial resolution to be a useful tool for SRS commissioning and quality assurance, including single‐isocenter multiple‐met modulated plans. The limitations of the device for some cases might be the size of the active area and inability to sample certain beams at their planned angles.

## CONFLICT OF INTEREST

SA is a graduate student supported by an SNC grant and VF is the PI on the project.
